# Study of Gonadal Hormones in Males With Liver Cirrhosis and Its Correlation With Child-Turcotte-Pugh and Model for End-Stage Liver Disease Scores

**DOI:** 10.7759/cureus.34035

**Published:** 2023-01-21

**Authors:** Bhumika Vaishnav, Rahul Tambile, Kartheek Minna, Srivatsav Addepalli, Aniruddh Wadivkar, Ruchitha Pailla, Nirali Thakkar, Snigdha Balem

**Affiliations:** 1 General Medicine, Dr. D. Y. Patil Medical College, Hospital and Research Centre, Pune, IND; 2 General Medicine, Sri Venkata Sai (SVS) Medical College, Mahbubnagar, IND

**Keywords:** model for end-stage liver disease, testosterone hormone, sex hormones, cirrhosis of the liver, child-pugh class

## Abstract

Background and aims: Liver cirrhosis influences gonadal hormone metabolism by multiple mechanisms and causes gonadal dysfunction. This study aimed to study sex hormones in males with cirrhosis and determine their correlation with prognostic scores.

Methods: An observational study was conducted between October 2019 and August 2021 in India. Sixty males with liver cirrhosis and 60 healthy age-matched controls were enrolled. Serum-free testosterone (T), estradiol (E2), follicle-stimulating hormone (FSH), luteinizing hormone (LH), and prolactin (Prl) were checked. Child-Turcotte-Pugh (CTP) and model for end-stage liver disease (MELD-Na) scores were calculated.

Results: Mean age of patients was 46.9±8.38 years. Forty-three were alcoholics. A total of 29 (48.33%) patients had low levels of free T. Cirrhotic males had lower testosterone and higher estradiol levels and lower T:E2 ratio compared to controls. Levels of luteinizing hormone, follicle-stimulating hormone, and prolactin were comparable. Lower testosterone was significantly associated with advancing age, alcoholism, duration of cirrhosis, loss of libido, and ascites. The higher the CTP scores, the lower the free testosterone levels and the higher the E2 levels. There was no significant association between low free testosterone levels and MELD-Na score.

Conclusions: Age, alcohol, duration of disease, and low albumin levels are risk factors for hypogonadism in cirrhosis. There was a significant positive correlation between low free testosterone levels and poor CTP scores.

## Introduction

The liver plays a fundamental role in the maintenance of free testosterone (FT) which is a biologically active form by synthesizing sex hormone binding globulin (SHBG) and albumin. The liver also maintains the endocrine homeostasis of the gonadal hormones by converting androgens into estrogens by aromatase enzyme and catabolizes 70% of the sex hormones by specific enzymes [1]. Patients with chronic liver diseases (CLD) have several endocrine dysfunctions, which include alterations in the functioning of the hypothalamic-pituitary-gonadal (HPG) axis. Changes in sex hormone concentrations have been found with high estrone and estradiol levels and low testosterone levels, which may partly account for these phenotypic changes [2]. Cirrhotic males often have a reduction in prostate size, loss of libido, impotence, oligospermia, infertility, and loss of body [3].

According to a study, low testosterone level in men with CLD is an independent predictor of mortality [4]. Measuring serum testosterone level is a simple test and can be used to improve the prognostic value of the model for end-stage liver disease (MELD) score. According to an Indian study done in 2009, secondary hypogonadism was more common in patients having advanced cirrhosis (Child-Pugh stage B and C) [5]. Very few studies have been done to assess all the reproductive hormones in males with cirrhosis and correlate them with prognostic scores like MELD and Child-Turcotte-Pugh (CTP). Hence the present study was conducted to assess all gonadal hormone levels in males with liver cirrhosis and aimed to find out whether any correlation exists between altered gonadal hormonal levels and MELD/CTP scores.

## Materials and methods

**Figure 1:**
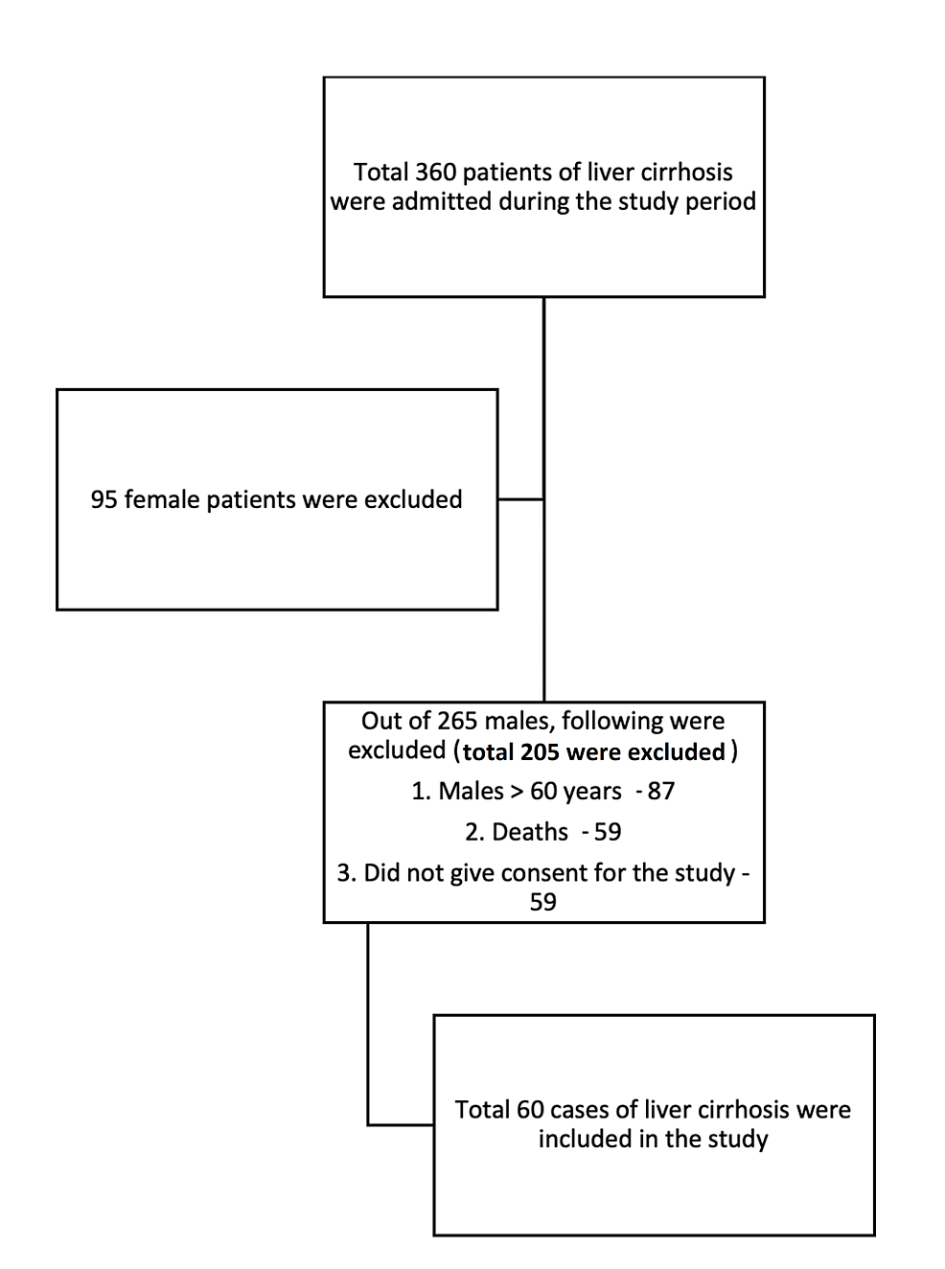
Algorithm for selection of patients for the study.

A cross-sectional, observational study was carried out on a total of 120 males between the age of 18 and 60 years. Sixty subjects with liver cirrhosis (henceforth known as cases) and 60 age-matched controls with healthy livers were studied. Figure 1 shows the patient selection algorithm. Total 360 patients with CLD were screened for enrolment in the study.

Inclusion and exclusion criteria

Patients with ultrasound of the abdomen showing liver parenchymal disease or a nodular and shrunken liver with coarse echotexture or biochemical evidence of liver disease of greater than six months duration with portal-hypertension on ultrasonography (USG) or upper gastrointestinal endoscopy (UGIE) were included in the study. Patients with age <18 years and ≥60 years, smokers, type 2 diabetes mellitus, HIV-positive status, chronic kidney diseases, on chronic steroid/hormonal therapy/spironolactone intake, history of prostatic or testicular malignancy, psychiatric disorders, and those who had undergone medical or surgical castration were excluded from the study.

Prior approval was taken from the institutional ethics committee (ethics approval no. IESC/PG/055/16) and written informed consent was obtained from all study participants. Both cases and controls were subjected to the following investigations: liver function tests (LFTs) (including total proteins, albumin, and globulin), prothrombin time with international normalized ratio (PT with INR), serum sodium (for model for end-stage liver disease {MELD-Na} score calculation), gonadal hormones (blood collected in the morning after 12-hour fasting state, serum was separated and stored at -80°C), serum-free testosterone (FT) by solid phase enzyme immunoassay method, serum estradiol (E2) by bi-directionally interfaced chemiluminescent immunoassay method, follicle-stimulating hormone (FSH), luteinizing hormone (LH), and prolactin levels by chemiluminescent microparticle immunoassay method. Following data collection, the Child-Turcotte-Pugh score was calculated on the basis of five parameters [6]. The patients were divided into Child classes A, B, or C if their scores were 5-6, 7-9, or greater than 10, respectively. MELD score was calculated using the following formula: 10 × ({0.957 × log_e _(creatinine)} + {0.378 × log_e _(bilirubin) + (1.12 × log_e _(INR)} + 6.43). MELD-Na score was further calculated using the below formula: (0.025 × MELD × {140-Na}) + 140.

The patients were divided into two groups - those with normal free testosterone levels (without gonadal dysfunction) and those with low free testosterone levels (with gonadal dysfunction). FT levels were considered to be low when the value was less than 4.5 pg/mL (defined low levels for the laboratory from where the test was ordered). Various statistical tests were applied to study the significance of the results.

Statistical analysis

Microsoft Excel 2010 was used to compile the collected data. The data was analyzed using OpenEpi Software version 2.3 and SPSS software version 15 (Armonk, NY: IBM Corp.). ANOVA, chi-square, Student's t-test, and Fisher’s exact tests were applied wherever applicable. All tests were two-tailed with a 95% confidence interval (p<0.05) considered statistically significant.

## Results

Table 1 shows the demographic parameters of the patients. The mean age of males with liver cirrhosis was 46.9±8.38 years with a mean weight of 67.05±10.01 kg and mean BMI of 25.5±3.51 kg/m^2^. The majority of the cases were in the age group of 41-50 years (41.67%). A total of 36.67% of subjects had a normal body mass index. A total of 11.67% of patients with CLD were not on treatment for the same. Thirty-nine out of 60 cirrhosis patients had a chronic alcohol addiction. Among the alcoholics, 76.75% were consuming cheap country liquor and 23.25% were consuming foreign liquor. Gynecomastia (96.66%) was the commonest sign followed by signs of feminization (91.66%) - change in voice, gynecoid fat distribution, and decreased hair growth. Loss of libido was found in 46 of 60 (76.66%) patients. Ascites and hepatic encephalopathy were present in 51.67% and 16.67% of patients, respectively. The mean Hb of the patients was low (8.66+2.3 g/dL), total bilirubin was 6.03+7.27 mg/dL and a majority of patients had direct hyperbilirubinemia (direct bilirubin: 5.66+6.04 mg/dL vs indirect bilirubin: 1.71+1.85 mg/dL). Serum aspartate transaminase was 113.7+113 U/dL and serum alanine transaminase was 75.2+150 U/dL. Mean serum albumin was low (2.8+0.6 g/dL) and international normalized ratio (INR) was high (1.3±0.4).

**Table 1:** Demographic parameters of patients with chronic liver disease (n=60). CLD: chronic liver diseases

Variables		Number of patients with CLD	
Age groups (years)	18-30	2 (3%)	
	31-40	10 (16.67%)	
	41-50	25 (41.67%)	
	51-60	23 (38.33%)	
BMI range	<18.5 (underweight)	2 (3.33%)	
	18.5-24.9 (normal)	22 (36.67%)	
	25-29.9 (overweight)	30 (50%)	
	30-34.9 (grade 1 obese)	6 (10%)	
	35-39.9 (grade 2 obese)	0	
	≥40 (morbidly obese)	0	
Duration of CLD	-	On treatment	Off treatment
	<5 years	37 (61.67%)	16 (26.67%)
	>5 years	5 (8.33%)	2 (3.33%)
Duration of alcohol addiction	-	Total number of alcoholics (n=43)	
	<5 years	9 (20.93%)	
	5-10 years	29 (67.44%)	
	>10 years	5 (11.63%)	
Signs and symptoms due to gonadal dysfunction	-	Number of patients (n=60)	
	Loss of libido	46 (76.66%)	
	Signs of feminization	55 (91.66%)	
	Gynecomastia	58 (96.66%)	
Etiology of cirrhosis	Alcohol	39 (65%)	
	Chronic hepatitis B infection	12 (20%)	
	Chronic hepatitis C infection	5 (8.33%)	
	Non-alcoholic fatty liver disease	4 (6.67%)	

Table 2 shows the mean values of gonadal hormones in cases and controls. Out of 60 males with liver cirrhosis, 29 (49.33%) had low FT levels and 31 (50.67%) had normal FT levels. Among the healthy controls, 52 patients had normal FT levels and eight had low FT levels. High E2 levels were found in 28 (46.67%) patients with cirrhosis and in 12 (20%) controls without cirrhosis. There was a statistically significant difference in mean FT and E2 levels (p< 0.05) between cases and controls (Student’s t-test), i.e., patients with liver cirrhosis had significantly lower FT values and higher E2 values as compared to healthy controls. In the majority of the 60 cirrhotic patients, serum prolactin (90%), serum LH levels (83.33%), and serum FSH levels (76.67%) were normal. Laboratory values for mean LH, FSH, and prolactin were not significantly different between cirrhosis patients and controls (p>0.05).

**Table 2:** Average gonadal hormonal levels in cases and controls. *P-value is significant. **P-value is non-significant.

Hormones	Cases with liver cirrhosis	Controls without cirrhosis	Normal laboratory values	Student’s t-test (p-Value)
Serum-free testosterone (pg/mL)	7.439±6.10	24.35±8.20	4.50-30.37	<0.05*
Serum estradiol (pg/mL)	43.53±15.13	13.42±3.78	11-44 (male)	<0.05*
Testosterone to estradiol ratio	0.27±0.4	1.67±0.24	1.7±0.12	<0.05*
Serum prolactin (ng/mL)	11.86±8.09	10.85±6.75	2.1-17	>0.05**
Serum follicle-stimulating hormone (mIU/mL)	8.71±12.60	8.54±7.20	1.4-18.1	>0.05**
Serum luteinizing hormone (mIU/mL)	6.34±6.09	6.45±3.25	1.5-9.0	>0.05**

Study subjects were further divided into two groups for the sake of statistical comparison - those with low FT values (with gonadal dysfunction) and those with normal FT levels. Table 3 shows various demographic and clinical parameters for patients with low and normal FT levels. Alcohol addiction, advancing age, and longer duration of cirrhosis were associated with low FT in the cases (p<0.05). There was no significant difference between the peripheral signs of liver cell failure in the two groups of patients, except for the loss of libido and ascites, which were significantly present in patients with low FT. On comparing the various laboratory parameters between the two groups, only low albumin level was associated with low testosterone (2.8±0.72 vs 3.5±0.4 g/dL) (p=0.0001). The remaining liver function tests were comparable between the two groups.

**Table 3:** Various demographic parameters in patients with low and normal free testosterone. *P-value is significant. **P-value is non-significant. CLD: chronic liver disease; INR: international normalized ratio; FET: Fisher’s exact test; STT: Student’s t-test, S: significant, NS: not significant

Demographic parameters	With low testosterone (n=29)	Without low testosterone (n=31)	Statistical tests (p-Value)
Age (years)	52.20±5.84	41.93±7.31	0.02 (STT)*
BMI (kg/m^2^)	24.73±3.49	26.20±3.43	0.1 (STT)**
Duration of CLD (years)	4.34±1.32	3.77±2.81	0.000213 (STT)*
Alcohol addiction present (n=39)	25	14	0.0011 (FET)*
Signs and symptoms			
Parotid gland enlargement	28 (96.55 %)	28 (90.32%)	0.6 (FET)**
Gynecomastia	28 (96.55 %)	30 (96.77 %)	1 (FET)**
Feminization	27 (93.10 %)	28 (90.32%)	1 (FET)**
Loss of libido	27 (93.10 %)	19 (61.29%)	0.005 (FET)*
Hepatic encephalopathy	8 (27.58%)	5 (16.12%)	0.28 (CST)**
Ascites	21 (72.41%)	9 (29.03%)	0.0007 (CST)*
Laboratory parameters			
Serum bilirubin (mg/dL)	6.97±7.39	7.76±7.39	0.68 (STT)**
Serum albumin (g/dL)	2.8±0.5	3.5±0.4	0.0001 (STT)*
INR (prothrombin time)	1.4±0.4	1.2±0.2	0.12 (STT)**

Of the 60 cirrhosis cases, 23 (38.33%) were in CTP class A, 26 (43.33%) were in CTP class B, and 11 (18.33%) were in CTP class C. Table 4 shows the relationship between various sex hormones and the CTP classes. The mean FT and E2 levels were statistically significantly different across the CTP classes (ANOVA test: p-values 0.01 and 0.001 for FT and E2, respectively). But differences among Prl, LH, and FSH across CTP classes were not statistically significant. Table 5 shows CTP scores in patients with low and normal FT. Patients with low FT levels had significantly higher CTP scores when compared to the patients with normal FT levels (p= 0.00001).

**Table 4:** Correlation between various gonadal hormones and CTP score. *P-value is significant. **P-value is non-significant. CTP: Child-Turcotte-Pugh

Gonadal hormones	CTP A (n= 23)	CTP B (n=26)	CTP C (n=11)	One-way ANOVA test (p-Value)
Serum-free testosterone (pg/mL)	9.99±6.27	5.32±5.78	5.77±5.75	0.01*
Serum estradiol (pg/mL)	35.21±15.25	50.24±14.79	52.65±15.3	0.001*
Serum prolactin (ng/mL)	12.49±8.34	9.61±8.43	12.83±8.79	0.37**
Serum follicular-stimulating hormone (mIU/mL)	9.43±8.55	14.04±12.97	9.85±13.39	0.4**
Serum luteinizing hormone (mIU/mL)	7.39±4.56	8.06±6.34	5.32±6.56	0.4**

**Table 5:** CTP score and class in patients with low and normal testosterone levels. CTP: Child-Turcotte-Pugh

CTP class	With low testosterone (n=29) (%)	With normal testosterone (n=31) (%)
A	3 (6.90)	20 (64.52)
B	20 (72.41)	6 (19.35)
C	6 (20.69)	5 (16.13)

Twelve out of 60 cases had a MELD-Na score of more than 20 and the remaining 48 patients had a MELD-Na score of less than 20. There was no significant difference in the mean MELD score between the cases with low T levels and cases with normal T levels (p>0.05). Thus, a relationship between T level and MELD-Na score was not established in this study.

## Discussion

Liver cell failure is the classic feature of cirrhosis where the healthy liver tissue is replaced by nodular fibrosis leading to the slowing or cessation of various liver functions [7]. Gonadal hormone dysfunction in cirrhosis depends on the degree of suppression of the hypothalamic-pituitary-gonadal axis (HPG axis), the cause of cirrhosis, and the degree of severity of liver disease [8,9].

There is secondary or mixed hypogonadism (both primary and secondary hypogonadism) in chronic liver diseases [10]. The mechanism of hypogonadism in cirrhosis is complex. In chronic liver diseases, there is a rise in SHBG, a fall in the production rate of testosterone, increased peripheral conversion of testosterone into estradiol (due to portosystemic shunting), and a rise in hepatic aromatase activity and affection of hypothalamic-pituitary-gonadal axis [11]. These changes lead to low free circulating testosterone levels and high total estradiol levels.

In the current study, the average FT levels were in the low normal range and E2 levels were in the high normal range in the cases group. FT:E2 ratio was significantly low in the cases group compared to the controls. Lower than-normal FT levels have been recorded previously [12]. The mean E2 levels, although higher than the control group, were within the normal range in the study. High estrogen level in cirrhosis is attributed to the increased peripheral conversion of androgens (androstenedione and testosterone) to estrogen. However, studies have proven that the estrone level (E1) rises more commonly compared to estradiol (E2) due to this peripheral conversion especially of testosterone [13,14]. Low FT:E2 ratio has been recorded in a few other studies. It has been a prevalent notion that gynecomastia in males with cirrhosis is due to the low FT:E2 ratio [15,16].

Mean levels of other gonadal hormones were in the normal range in the current study same as few other studies [17,18]. As discussed earlier, hypogonadism in liver cirrhosis is secondary and multifactorial. The fact that S. FSH and LH were in the normal range with a low FT level indicates the dysfunction of the hypothalamic-pituitary-gonadal axis in men with cirrhosis [17,18]. Loss of libido was present in three-quarters of the patients. Sexual dysfunction in men with cirrhosis of liver was found in 61% of patients in a study by Jensen et al. [19]. A review article by Karagiannis et al. mentions that 50% of patients with liver cirrhosis show signs of feminization, testicular atrophy, decreased libido, reduced secondary sex hair, and gynaecomastia associated with reduced spermatogenesis and peritubular fibrosis [20]. Loss of libido may be due to aging, erectile dysfunction, lack of sexual desire, and chronic disorders like diabetes mellitus and kidney and liver failure. We excluded geriatric patients, patients with diabetes mellitus, chronic kidney diseases, or psychiatric illnesses from the study. Hence, the loss of libido found in the patients may be attributed primarily to liver dysfunction. Chronic liver diseases cause profound physical and psychological changes in an individual leading to loss of libido. Low circulating FT, high E2, and chronic alcohol addiction as was found in the study may be contributing to this. Studies have concluded that sexual dysfunction in cirrhosis patients does not improve significantly post Liver transplant and may be due to certain irreversible changes in the gonadal hormone metabolic pathways and testicular atrophy [21].

Chronic alcohol addiction of more than five years duration was responsible for liver disease in 65% of cases and was significantly associated with low FT levels in the current study. Gonadal dysfunction in alcoholics may be due to the depressant effect of alcohol itself, alcohol-related liver disease, or due to psychological factors [22]. Alcohol affects testosterone levels by multiple mechanisms - it suppresses the HPG axis in men and directly reduces its production at the testicular level [23].

In the current study, advancing age, longer duration of CLD, chronic alcohol addiction, and low albumin levels were statistically significantly associated with low testosterone levels (p<0.05). A similar study showed that advancing age, low serum albumin, high total bilirubin, and prothrombin time were significantly associated with secondary hypogonadism and low testosterone values [24]. The findings of the current study did not match this.

There is a decline in free testosterone levels with aging due to a rise in SHBG levels [25]. All chronic diseases including cirrhosis of liver and chronic alcohol addiction affect the function of the Leydig cells in the testis, cause the release of cytokines (which increase the peripheral conversion of androgens to estrogens), erectile dysfunction, and testicular atrophy [1]. Thus with increasing duration of the liver malfunction and long-standing alcohol addiction, the fall in testosterone levels and hypogonadism become pronounced and may not be reversible even after LT. The low FT level was significantly associated with the worsening of the CTP score (p<0.05). This finding corroborated the findings of a few other studies [12,18]. However, there was no correlation between the LH, FSH, and prolactin levels and the worsening of the CTP score.

The median MELD-Na score in the present study was 14. The MELD-Na score was not different between cirrhosis patients with low and normal FT levels (p>0.05). A study by Grossmann et al. had a median MELD score of 16 (IQR 25th to 75th percentile) [17]. The MELD score was not different between men with and without hypogonadism which was similar to our findings. Another study also showed no association between the MELD score and gonadal hormones (LH, FSH, and prolactin) except estradiol [26]. Another study concluded that FT can be a novel prognostic marker for increased mortality and the need for LT independent of the MELD score [27].

Small sample size, evaluation of free testosterone at a single point in time, and bias in patient selection were the few limitations of the study.

## Conclusions

Free testosterone level is low and estradiol level is high in patients with liver cirrhosis. Long duration of the disease, advancing age, alcoholism, and hypoalbuminemia are associated with low FT. The FT level correlates with poor Child-Pugh score but not with the MELD-Na score. Sexual dysfunction and loss of libido in cirrhosis are multifactorial and may be associated with poorer prognosis. Further studies about the effect of testosterone replacement therapy in advanced cirrhosis cases are warranted.
